# Small-quantity, lipid-based nutrient supplements provided to women during pregnancy and 6 mo postpartum and to their infants from 6 mo of age increase the mean attained length of 18-mo-old children in semi-urban Ghana: a randomized controlled trial^1^,^2^

**DOI:** 10.3945/ajcn.116.134692

**Published:** 2016-08-17

**Authors:** Seth Adu-Afarwuah, Anna Lartey, Harriet Okronipa, Per Ashorn, Janet M Peerson, Mary Arimond, Ulla Ashorn, Mamane Zeilani, Stephen Vosti, Kathryn G Dewey

**Affiliations:** 3 Department of Nutrition and Food Science, University of Ghana, Legon, Accra, Ghana;; 4 Center for Child Health Research, University of Tampere School of Medicine and Tampere University Hospital, Tampere, Finland;; 5 Program in International and Community Nutrition, Department of Nutrition, University of California, Davis, Davis, CA; and; 6 Nutriset S.A.S., Malaunay, France

**Keywords:** lipid-based nutrient supplements, child growth, home fortification, multiple micronutrients, supplementation

## Abstract

**Background:** Childhood stunting usually begins in utero and continues after birth; therefore, its reduction must involve actions across different stages of early life.

**Objective:** We evaluated the efficacy of small-quantity, lipid-based nutrient supplements (SQ-LNSs) provided during pregnancy, lactation, and infancy on attained size by 18 mo of age.

**Design:** In this partially double-blind, individually randomized trial, 1320 women at ≤20 wk of gestation received standard iron and folic acid (IFA group), multiple micronutrients (MMN group), or SQ-LNS (LNS group) daily until delivery, and then placebo, MMNs, or SQ-LNS, respectively, for 6 mo postpartum; infants in the LNS group received SQ-LNS formulated for infants from 6 to 18 mo of age (endline). The primary outcome was child length by 18 mo of age.

**Results:** At endline, data were available for 85% of 1228 infants enrolled; overall mean length and length-for-age *z* score (LAZ) were 79.3 cm and −0.83, respectively, and 12% of the children were stunted (LAZ <−2). In analysis based on the intended treatment, mean ± SD length and LAZ for the LNS group (79.7 ± 2.9 cm and −0.69 ± 1.01, respectively) were significantly greater than for the IFA (79.1 ± 2.9 cm and −0.87 ± 0.99) and MMN (79.1 ± 2.9 cm and −0.91 ± 1.01) groups (*P* = 0.006 and *P* = 0.009, respectively). Differences were also significant for weight and weight-for-age *z* score but not head or midupper arm circumference, and the prevalence of stunting in the LNS group was 8.9%, compared with 13.7% in the IFA group and 12.9% in the MMN group (*P* = 0.12). In analysis based on actual supplement provided at enrollment, stunting prevalences were 8.9% compared with 15.1% and 11.5%, respectively (*P* = 0.045).

**Conclusion:** Provision of SQ-LNSs to women from pregnancy to 6 mo postpartum and to their infants from 6 to 18 mo of age may increase the child’s attained length by age 18 mo in similar settings. This trial was registered at clinicaltrials.gov as NCT00970866.

## INTRODUCTION

Poor linear growth of children is a major problem in developing countries, where an estimated 28% of children <5 y of age are stunted (1). Childhood stunting is associated with several negative consequences, including impaired cognitive development (2–4) and increased risk of death from infectious diseases (5). Although stunted children may experience catch-up growth (6), their mean height deficit usually persists into adulthood (7, 8), a situation that is linked to low productivity (9) and increased risk of noncommunicable diseases (3). Because of the deleterious short- and long-term consequences of stunting, its prevention has become a key national and international priority (10, 11).

The process of stunting is most marked during the first 12–18 mo (12) of life, but it usually begins in utero (4) or soon after birth and may continue until 24 mo of age (13). It is logical, therefore, that interventions to reduce childhood stunting may need to include both the prenatal period and the first 1–2 postnatal years. In a previous landmark study by the Institute of Nutrition of Central America and Panama conducted in the 1970s (14), a comprehensive approach involving supplementation during pregnancy, lactation, and early childhood was associated with greater length gain in children and long-term effects on cognitive development and adult stature. However, since the Institute of Nutrition of Central America and Panama trial (14), there have been very few efforts to evaluate the impact of comprehensive nutritional supplementation given during most of the “first 1000 days” on stunting.

The International Lipid-Based Nutrient Supplements (iLiNS)^7^ Project developed small-quantity (20 g/d or 118 kcal/d), lipid-based nutrient supplements (SQ-LNSs) for pregnant and lactating women and for infants (15), following initial work with infants in Ghana (16) and Malawi (17), with the purpose of enriching local diets with micronutrients and essential fatty acids (EFAs). SQ-LNSs were designed to address concerns that in many populations the total energy content of the usual diet may be adequate, but the micronutrient (18) and EFA (19) contents may be low. In the iLiNS-DYAD trials carried out in Ghana and Malawi by the International Lipid-based Nutrient Supplement study group, in which mother-child dyads were enrolled, the efficacy of SQ-LNSs provided to women during pregnancy and the first 6 mo postpartum and to their offspring from 6 to 18 mo of age was tested by using a similar study design. In Malawi, this supplementation strategy may have had a modest impact on birth length among selected subgroups of women (20), but it did not affect child growth by 18 mo of age (21) when compared with 2 control groups, namely iron and folic acid (IFA) provided during pregnancy only or a multiple micronutrient (MMN) supplement with most of the same micronutrients as the SQ-LNSs provided during pregnancy and the first 6 mo postpartum. For the Ghana study, we previously reported on the first of the 2 primary hypotheses (22) and showed that infants born to mothers who received prenatal supplementation with SQ-LNSs compared with IFA had higher birth weight, and among those born to primiparous women, infants also had higher birth length and head circumference than both control groups (IFA and MMN). This article reports on the second hypothesis, namely that child growth at 18 mo of age would be greater in children whose mothers received SQ-LNSs and who themselves were supplemented with SQ-LNSs than in those whose mothers were in the 2 control groups.

## METHODS

### Study setting, design, participants, and enrollment

Details of the study setting, design, participants, and blinding schemes were reported previously (22). Briefly, the study was conducted in several adjoining semi-urban communities (Somany-Odumasi-Kpong area) in the Yilo Krobo and the Lower Manya Krobo districts ∼70 km north of Accra. It was designed as a partially double-blind, parallel, individually randomized, controlled trial with 3 equal-size groups. Pregnant women attending usual antenatal clinics in the 4 main health facilities in the area were included if they were ≥18 y old and at ≤20 wk of gestation based on the information available at the time. They were excluded if any of the following applied: not residing in the area, intention to move within the next 2 y, milk or peanut allergy, participation in another trial, HIV infection, asthma, epilepsy, tuberculosis, any malignancy, or unwillingness to sign or thumbprint the relevant consent forms, receive field workers, or take the study supplement.

After baseline assessments, eligible women were randomly assigned to receive one of 3 treatments: *1*) 60 mg Fe plus 400 μg folic acid during pregnancy and 200 mg Ca serving as a placebo for the first 6 mo postpartum (hereafter, IFA supplement or group); *2*) MMN capsule containing 18 vitamins and minerals (including 20 mg Fe) during pregnancy and for the first 6 mo postpartum, (hereafter, MMN supplement or group); and *3*) SQ-LNSs with similar micronutrients as the MMN supplement in addition to calcium, phosphorus, potassium, and magnesium as well as energy (118 kcal/d) and macronutrients (e.g., protein and EFAs) during pregnancy and for the first 6 mo postpartum, followed by supplementation for their offspring from 6 to 18 mo of age by using SQ-LNSs designed for infants (hereafter, LNS supplement or group). The IFA and MMN supplements served as controls because at the time of the study, IFA was the standard WHO nutritional supplementation for pregnant women (23), and available evidence suggested that prenatal supplementation with MMN supplements promoted fetal growth (24–26).

The study statistician at University of California, Davis developed group allocations with the use of a computer-generated (SAS version 9.3; SAS Institute) randomization scheme in blocks of 9. At each enrollment, the study nurse offered sealed, opaque envelopes bearing group allocations, 9 envelopes at a time, and the woman picked one to reveal the allocation. Allocation information was kept securely by the field supervisor and the study statistician only. Enrollment was completed between December 2009 and December 2011. The study protocol was approved by the ethics committees of the University of California, Davis; the Ghana Health Service; and the University of Ghana Noguchi Memorial Institute for Medical Research, and was registered on clinicaltrials.gov as NCT00970866. A 5-member independent Data and Safety Monitoring Board monitored the incidence of serious adverse events (SAEs).

### Study supplements and blinding

The micronutrient contents of the maternal supplements are reported elsewhere (22) as was the rationale underlying the concentrations of the nutrients used in all the supplements, including SQ-LNSs for infants (15). Apart from iron, which was kept at 20 mg/d in the MMNs and SQ-LNSs for women, the vitamin and mineral contents were either 1 time or 2 times the Recommended Dietary Allowance for pregnancy or, in a few cases, the maximum amount that could be included in the supplement given technical and organoleptic constraints. The nutrients in the SQ-LNS for infants (20 g/d) were energy (118 kcal), protein (2.6 g), fat (9.6 g), linoleic acid (4.46 g), α-linolenic acid (0.58 g), vitamin A (400 μg retinol equivalents), thiamin (0.3 mg), riboflavin (0.4 mg), niacin (4 mg), vitamin B-6 (0.3 mg), vitamin B-12 (0.5 μg), vitamin C (30 mg), vitamin D (5 μg), vitamin E (6 mg), vitamin K (30 μg), folic acid (80 μg), pantothenic acid (1.8 mg), iron (6 mg), zinc (8 mg), copper (0.34 mg), calcium (280 mg), phosphorus (190 mg), potassium (200 mg), magnesium (40 mg), selenium (20 μg), iodine (90 μg), and manganese (1.2 mg).

The IFA and MMN supplements were provided as capsules in blister packs, and the LNS supplements for women were in 20-g sachets and those for infants in 10-g sachets (2 given/d). All supplements were intended for daily consumption, the IFA and MMN with water after a meal, 1 capsule/d. The LNS supplements for women were mixed with any prepared food, 1 sachet/d, and LNS supplements for infants were mixed with complementary foods, 1 sachet at a time, on 2 different occasions during the day. To maintain blinding, 2 individuals independent of the study placed color-coded stickers (3 different colors for IFA and 3 for MMN supplements), which also bore the letters P or L (indicating consumption during pregnancy or lactation, respectively) behind the blister packs, so that the capsules were known to the study team and participants only by the colors of the stickers. It was not possible to blind study workers and participants to the capsules (IFA and MMN supplements) compared with the LNS supplements because of their apparent differences, but laboratory staff, anthropometrists, and data analysts had no knowledge of group assignment until all preliminary analyses had been completed.

### Procedures

At baseline, we collected women’s sociodemographic information, determined gestational age (GA) by ultrasound biometry (Aloka SSD 500; Hitachi), except for a few who had ultrasound information at the time of screening, and assessed women’s anthropometric status (standard procedures), hemoglobin (HemoCue AG; Wetzikon) and malaria parasitemia (Vision Biotech; South Africa) (22). Immediately after enrollment, the study nurse gave each woman a 2-wk supply of assigned supplement (IFA or MMN, bearing the letter P, or LNS for women), with a description of mode of consumption and a standard nutrition message (“Do not forget to eat meat, fish, eggs, fruits, and vegetables whenever you can; you still need these foods even as you take the supplement we have given you.”). Thereafter, field workers visited women in their homes biweekly until delivery and delivered fresh supplies of supplements as well as monitored morbidity and supplement intakes during each visit (22).

At delivery field workers immediately delivered a new supply of LNSs or color-coded capsules bearing the letter L and thereafter visited women and their infants each week. All live-born singleton infants were automatically enrolled. For twins (*n* = 22), only one infant was randomly selected, and the selected infant was enrolled if he or she was born alive. Stillborn infants were not enrolled. Within 48 h after delivery or between 3 and 14 d after delivery for 105 infants (9%), trained anthropometrists recorded the infants’ date of birth and measured the infants’ birth weight to the nearest 20 g (Seca 383; Seca), length to the nearest 0.1 cm (Seca 416; Seca), and head and midupper arm circumferences (MUAC) to the nearest 0.1 cm (Shorr Productions) by using procedures described by WHO (27). These measurements were performed in duplicate, unless the second measurement differed from the first by more than a predefined tolerable amount (0.1 kg for weight and 0.5 cm for length, head circumference, and MUAC), in which case a third measurement was taken.

At every other weekly home visit until 6 mo postpartum, women received a fresh supply of supplements, and their supplement consumption was monitored. Data on morbidity and SAEs were collected for women biweekly until 6 mo postpartum and for infants weekly until 18 mo of age. Women (and their husbands, if present) were told to call the project office or field supervisor whenever there was any or suspected SAE (e.g., death or hospitalization) in a study woman or infant, and therefore field workers following-up on such calls also collected data on SAEs between the weekly visits. At 3, 6, 12, and 18 mo of age, children were brought to the laboratory, and the anthropometric measurements carried out at birth were repeated. All anthropometrists were standardized according to WHO standards (27) shortly before data collection began and then every 6 mo thereafter.

At the weekly home visit after the anthropometric measurements at 6 mo of age, field workers delivered the following standard nutrition message to all mothers, which was also repeated by the study nurse during the anthropometric measurements at 12 mo of age: “breastfeed your baby as you did before 6 mo of age; do not forget to give your baby other foods such as meat, fish, eggs, fruits, and vegetables whenever you can because your baby still needs these foods.” If a mother was in the LNS group, the fieldworker modified the last part of the message to say, “Your baby still needs these foods even if you give him/her the infants’ LNSs.” Mothers of infants who had not yet received any complementary foods were told to “start giving complementary foods to the infants as soon as possible, because breast milk alone is not enough for the baby after he or she is 6 mo old.” To mothers in the LNS group, fieldworkers gave a 1-wk supply of infants’ LNSs along with the instruction to mix the entire content of one sachet with 2–3 tablespoons (30–45 mL) of any food for the infants before feeding additional foods if the infants desired, 2 times/d. Mothers in the LNS group who had not yet started feeding complementary foods to their infants were told to do so for at ≥3 d before feeding LNSs to the infants. Mothers in the LNS group received a fresh supply of infants’ LNSs every week thereafter until the infants exited the study at 18 mo of age.

Primary outcomes were infants’ length (cm) and length-for-age *z* score (LAZ) at 18 mo of age. Secondary outcomes were infants’ weight (kg); head circumference (cm); MUAC (cm); *z* scores for weight-for-age (WAZ), weight-for-length (WLZ), and head circumference-for-age (HCZ); stunting; underweight; wasting; small head circumference at 18 mo of age; growth from 0 (birth) to 18 mo of age; and incidence of SAEs from 0 to 18 mo of age.

### Sample size and data analysis

An effect size (Cohen’s *d*) of 0.3 (small-to-moderate effect size) (28) was the basis for all sample size calculations in the iLiNS-DYAD-Ghana study. This produces the same target sample size regardless of the outcome being considered, because it is independent of the units of measurement. Thus, our sample size was based on detecting an effect size of 0.3 between any 2 groups for any continuous variable at any time point (e.g., 18 mo of age), with a 2-sided 5% test and 80% power.

We enrolled 1320 pregnant women into the study, as described previously (22). Because of the temporary mislabeling of IFA and MMN capsules described previously (22), 170 women initially assigned to the IFA group inadvertently received the MMN capsule either throughout pregnancy (*n* = 85) or during part of pregnancy (*n* = 85) before receiving the intended IFA capsule, and another 170 women initially assigned to the MMN group received the IFA capsule either throughout pregnancy (*n* = 78) or during part of pregnancy (*n* = 92) before receiving the intended MMN capsule. In this current analysis, we elected to include all of the children enrolled (including those whose mothers temporarily received the unintended capsule) because no children received any unintended supplements themselves, and the consumption of the unintended supplements by the relatively small number of women during pregnancy likely had little if any impact on child growth in the entire sample by 18 mo of age. With a total sample size of ∼1043 children (∼348/group) for whom anthropometric data were available at 18 mo of age, we had 97% power to detect an effect size of 0.3 between any 2 groups for any continuous outcome, and 80% power to detect an RR of >2.1 for stunting, >2.4 for underweight, >4.0 for wasting, and >2.0 for small head circumference.

We posted the statistical analysis plan (www.ilins.org) before analysis. Statistical analysis was performed by using SAS version 9.3 on an intention-to-treat basis. That is, children were included in the analysis regardless of adherence to treatment. To address the protocol violation associated with the consumption of mislabeled capsules by some women during pregnancy, we analyzed our data by using 2 scenarios: in the first, intervention groups were based on the supplement that women were intended to receive when they were enrolled, and in the second, intervention groups were based on the supplement that women actually received when they were enrolled. The latter scenario is consistent with our previous publication reporting the birth outcomes of this trial (22). In addition, we performed a secondary analysis by using a 2-group comparison in which the IFA and MMN groups were combined.

We summarized the background characteristics of women at enrollment as means ± SDs for continuous variables, or number of participants and percentages for categorical variables by using the assignment of women based on supplements received at enrollment to be consistent with our previous publication (22). We calculated a household assets index and a housing index as proxy indictors for household socioeconomic status as well as the household food insecurity access score (22). Adherence to maternal supplement intake during pregnancy and postpartum was calculated as the percentage of days from enrollment to delivery and from delivery to 6 mo postpartum, respectively, when the supplements were reportedly consumed by women. We calculated adherence during pregnancy and postpartum separately in case these differed, and compared them between the 3 groups by using assignment based on supplements received at enrollment. For infants in the LNS group, adherence was calculated as the percentage of days from 6 to 18 mo of age when LNSs were reportedly added to the child's food. For growth measurements, we used the mean of duplicate measurements, or in the case of triplicate measurements, we used the mean of the 2 closest values. We determined LAZs, WAZs, WLZs, and HCZs as described by WHO (29), and considered values <−2.0 indicative of stunting, underweight, wasting, and small head circumference, respectively.

We evaluated the impact of the intervention by comparing continuous and binary outcomes by 18 mo of age between the 3 groups for each of the 2 analysis scenarios mentioned above. In a secondary analysis to examine postnatal growth only, we compared the change in continuous growth outcomes from birth to 18 mo of age (defined as the difference between the 18-mo and birth values of the outcome). These analyses were accomplished by using linear (continuous outcomes) and logistic (binary) regression models (SAS; PROC GLIMMIX), with Tukey-Kramer adjustment for multiple comparisons. Along with the group comparisons, we estimated all pairwise differences in means (continuous outcomes) and RRs (binary outcomes) with their 95% CIs and *P* values. RRs were calculated by using Poisson regression (30). We performed these analyses first without any covariates (unadjusted) and then with prespecified covariates (adjusted) if the covariates were significantly associated with the outcome at a 10% level of significance in a correlation analysis. The potential covariates were maternal age, height, BMI, education, GA at enrollment, primiparity, season at enrollment, and baseline anemia status; proxy indicators for household socioeconomic status, namely assets, food insecurity, and housing scores; and child sex. For the binary outcomes, the covariate-adjusted percentages were generated by using the technique described by Kleinman and Norton (31). In a separate exploratory analysis, we examined the mean LAZ at 0 (birth), 3, 6, 12, and 18 mo (SAS) to determine whether the group differences that were present at birth persisted during the postnatal period.

We evaluated potential interaction of intervention group with the prespecified maternal, household, and child variables listed above. When an interaction was significant (*P* < 0.10), we performed subgroup analysis by including an interaction term between treatment and the effect modifier in the ANCOVA or logistic regression model. For continuous effect modifiers, we created, using data from all participants, a linear regression model with which we predicted the values of the outcome at the 10th and 90th percentiles of the effect modifier distribution. Each effect modifier was considered separately in the models to reduce collinearity.

Finally, we used logistic regression (binary variables) and ANOVA (continuous variables) models to evaluate the occurrence of SAEs in women during pregnancy and lactation and in infants from birth until exit at 18 mo of age. These SAEs were defined to include deaths, hospitalizations (at least overnight stay in the hospital because of illness), congenital abnormalities, and life-threatening conditions requiring an immediate hospital visit. The SAEs recorded from enrollment to delivery (including miscarriage and stillbirths) were previously reported (22).

## RESULTS

**FIGURE 1:**
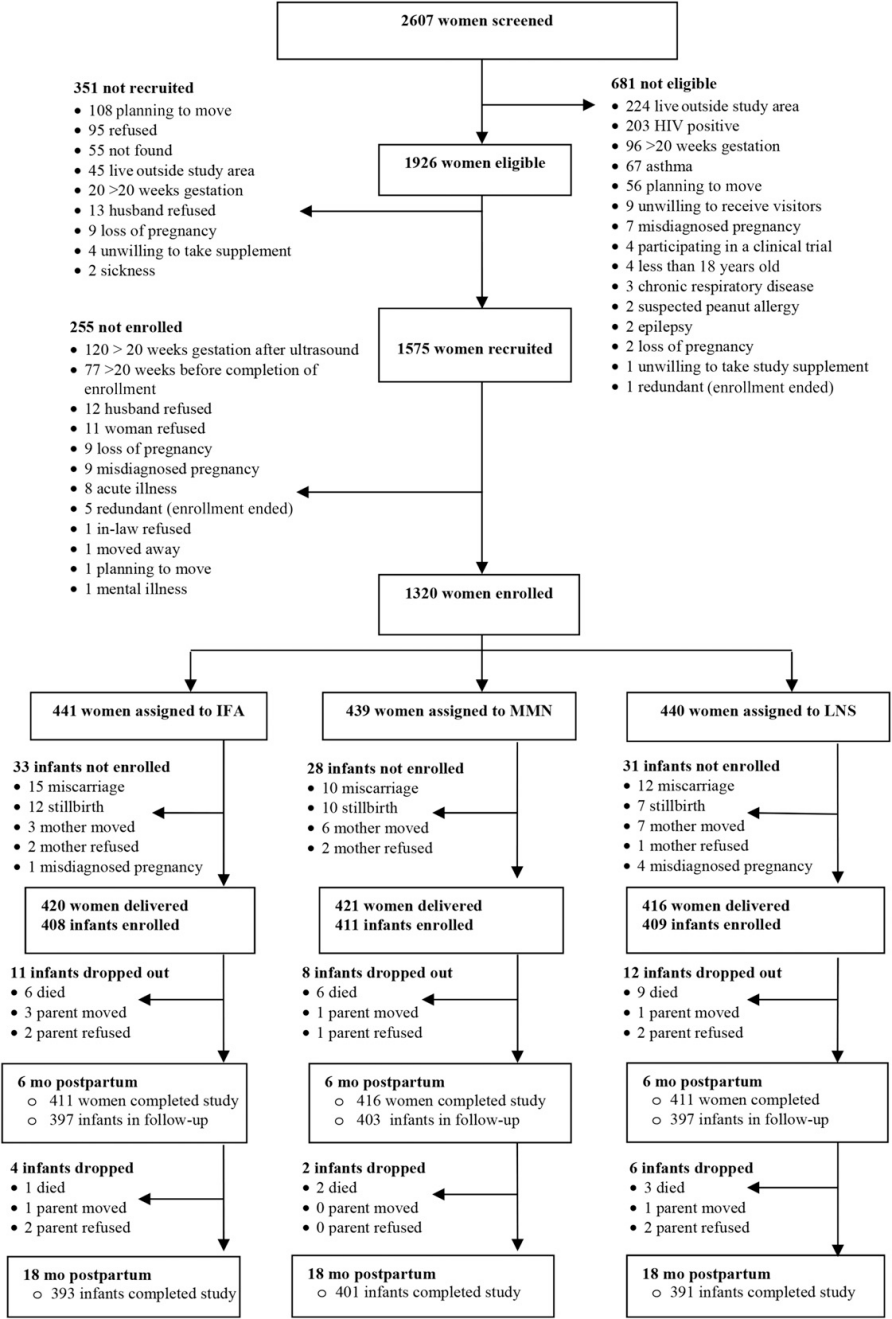
Study profile of the randomized trial of daily IFA (pregnancy only), MMN (pregnancy and lactation), and LNS (pregnancy, lactation, and infancy) supplementation in a semi-urban setting in Ghana. IFA, iron and folic acid; LNS, small-quantity lipid-based nutrient supplement; MMN, multiple micronutrient.

Data collection took place from December 2009 to March 2014. We screened 2607 women (22), of whom 681 were not eligible mainly as a result of living outside of the study area, being HIV positive, being at >20 gestational weeks, being asthmatic, or planning to move out of the area (**Figure 1**). Of the eligible women, 351 were not recruited (planning to move, refused, not found, etc.), whereas 255 were recruited (signed consent form) but not enrolled (did not participate in baseline assessment and random assignment) mainly because their GA advanced to >20 wk before enrollment could be completed. A total of 1320 pregnant women were enrolled in the 3 groups. On average, these women were in their mid-20s, had ∼7 y of formal education, had a GA of ∼16 wk, and were balanced across groups on all the background characteristics listed in **Table 1**. The total follow-up days during which women in the IFA and MMN groups were exposed to the unintended supplements was estimated at 37,470 woman-days (details not shown), and for the 880 women assigned to those 2 groups, the total follow-up days during the study period (pregnancy and 6 mo postpartum) was 293,059 woman-days. Therefore, the unintended exposure was 13% of woman-days for the IFA and MMN groups during the study.

**TABLE 1:** Background characteristics of women (*n* = 1320) who participated in a randomized trial of daily IFA (pregnancy only), MMN (pregnancy and lactation), and LNS (pregnancy, lactation, and infancy) supplementation in a semi-urban setting in Ghana by intervention group at enrollment^1^

Background characteristics^2^	IFA (*n* = 441)	MMN (*n* = 439)	LNS (*n* = 440)
Age, y	26.5 ± 5.2 (441)	26.7 ± 5.7 (439)	26.9 ± 5.6 (440)
Formal education, y	7.8 ± 3.6 (441)	7.6 ± 3.5 (439)	7.6 ± 3.9 (440)
Gestational age at enrollment, wk	16.2 ± 3.3 (438)	16.0 ± 3.2 (438)	16.1 ± 3.3 (435)
Asset index^3^	0.05 ± 1.01 (433)	0.05 ± 0.99 (431)	−0.09 ± 1.00 (432)
Housing index^3^	0.05 ± 0.98 (433)	−0.03 ± 1.02 (431)	−0.01 ± 1.00 (432)
Household Food Insecurity Access score^4^	2.8 ± 4.6 (436)	2.4 ± 4.1 (429)	2.6 ± 4.0 (432)
Estimated prepregnancy BMI,^5^ kg/m^2^	24.4 ± 4.2 (431)	24.3 ± 4.5 (430)	24.8 ± 4.5 (430)
Married or cohabiting, *n*/*N* (%)	406/441 (92.1)	413/439 (94.1)	405/440 (92.0)
Primiparous women, *n*/*N* (%)	162/441 (36.7)	137/439 (31.2)	147/440 (33.4)
Tested positive for malaria,^6^ *n*/*N* (%)	40/441 (9.1)	39/438 (8.9)	54/440 (12.3)

1 Values are mean ± SDs (*n*) unless otherwise indicated. IFA, iron and folic acid; LNS, small-quantity lipid-based nutrient supplement; MMN, multiple micronutrient.

2 Background characteristics were analyzed by using groups based on supplements received at enrollment. *n*/*N* = number of participants whose response was “yes” for the variable in question/*n* of participants analyzed for the variable in question.

3 Proxy indicators for household socioeconomic status; higher values represent higher socioeconomic status.

4 Household Food Insecurity Access is a proxy indicator for household food insecurity (58); higher values represent higher food insecurity.

5 Estimated prepregnancy BMI was calculated from estimated prepregnancy weight (based on polynomial regression with gestational age, gestational age squared, and gestational age cubed as predictors) and height at enrollment.

6 Rapid Diagnostic Test (Clearview Malarial Combo).

Of 1257 deliveries (including 22 sets of twins), 1228 infants were enrolled at birth, and 1185 completed the study by 18 mo of age. Women who were enrolled but whose infants did not complete the study (10%) did not differ in most background characteristics from those whose infants did, except the former were older (27.8 compared with 26.6 y) and had lower mean GA (15.5 compared with 16.2 wk) at enrollment. Reported adherence (percentage of days supplements were reportedly consumed) to supplement intake in women during pregnancy and postpartum was significantly lower (*P* < 0.001 in both cases) for the LNS group (69.3% ± 24.1% and 67.4% ± 26.5%, respectively) than either the IFA (76.8% ± 17.9% and 73.8% ± 22.5%) or MMN (73.6% ± 20.5% and 72.9% ± 24.1%) group. Reported adherence in infants supplemented with LNSs during 6–18 mo of age was 73.5%.

We obtained anthropometric data from ∼94%, 85%, 86%, 82%, and 85% of enrolled infants at 0 (birth), 3, 6, 12, and 18 mo of age, respectively. For the primary outcome (length) data from 1039 children (IFA: *n* = 350; MMN: *n* = 342; and LNS: *n* = 347) were analyzed at 18 mo of age. At none of these time points did the 3 groups differ significantly in the proportion of children from whom length data were obtained. At 18 mo of age, overall mean ± SD length, weight, head circumference, and MUAC of children were 79.3 ± 2.9 cm, 9.7 ± 1.2 kg, 45.3 ± 1.3 cm, and 14.2 ± 1.0 cm, respectively, corresponding to mean LAZ of −0.83 ± 1.01, WAZ of −0.80 ± 1.03, WLZ of −0.56 ± 1.01, and HCZ of −1.1 ± 0.88, respectively. In all, 12% of children were stunted, 12% were underweight, 7% were wasted, and 15% had small head circumference.

### Main group comparisons

**Table 2** presents the unadjusted continuous anthropometric outcomes for infants by 18 mo of age, by intervention group according to the analytic scenarios described in the Methods section. In the comparison of intervention groups based on intended supplement at enrollment, the mean length of infants in the LNS group was 0.58 cm (95% CI: 0.07, 1.10 cm) greater than that of infants in the IFA group, which corresponded to 0.18 (95% CI: 0.00, 0.36) in LAZ, and 0.64 cm (95% CI: 0.12, 1.16 cm) greater than that of infants in the MMN group, which corresponded to 0.22 (0.04, 0.40) in LAZ. The mean weight of infants was 0.24 kg (95% CI: 0.03, 0.46 kg) greater in the LNS group than in the MMN group, which corresponded to 0.21 (0.02, 0.39) in WAZ, but they did not differ significantly between the LNS and IFA groups. In the comparisons based on the supplements women received at enrollment, infants in the LNS group were 0.85 cm (95% CI: 0.33, 1.37 cm) longer, corresponding to 0.28 (95% CI: 0.10, 0.46) in LAZ, and 0.30 kg (95% CI: 0.08, 0.51 kg) heavier, corresponding to 0.24 (95% CI: 0.05, 0.42) in WAZ, than those in the IFA group, but they did not differ significantly in length or weight from those in the MMN group. At 18 mo of age, infants in the 3 groups (by using either analytic scenario) did not differ in mean head circumference, HCZ, or MUAC, and those in the IFA and MMN groups also did not differ significantly in any of those continuous growth outcomes. Results were generally similar when adjusted for selected covariates (data not shown).

**TABLE 2:** Unadjusted continuous anthropometric outcomes by 18 mo of age of infants of women (*n* = 1320) who participated in a randomized trial of daily IFA (pregnancy only), MMN (pregnancy and lactation), and LNS (pregnancy, lactation, and infancy) supplementation in a semi-urban setting in Ghana, by intervention group^1^

	Intervention group based on intended supplement at enrollment				Intervention group based on supplements received at enrollment			
Outcome variable	IFA (*n* = 441)	MMN (*n* = 439)	LNS (*n* = 440)	*P*^2^	IFA (*n* = 441)	MMN (*n* = 439)	LNS (*n* = 440)	*P*^2^
Length, cm	79.1 ± 2.9^b^ (353)	79.1 ± 2.9^b^ (342)	79.7 ± 2.9^a^ (348)	0.006	78.9 ± 2.8^b^ (340)	79.3 ± 3.0^a,b^ (355)	79.7 ± 2.9^a^ (348)	0.001
LAZ	−0.87 ± 0.99^b^ (350)	−0.91 ± 1.01^b^ (342)	−0.69 ± 1.01^a^ (347)	0.009	−0.97 ± 0.97^b^ (337)	−0.81 ± 1.01^a,b^ (355)	−0.69 ± 1.01^a^ (347)	0.001
Weight, kg	9.70 ± 1.20^a,b^ (353)	9.63 ± 1.20^b^ (343)	9.87 ± 1.23^a^ (348)	0.026	9.58 ± 1.17 ^b^ (340)	9.75 ± 1.22 ^a,b^ (356)	9.87 ± 1.23^a^ (348)	0.006
WAZ	−0.81 ± 1.01^a,b^ (350)	−0.90 ± 1.04^b^ (343)	−0.69 ± 1.04^a^ (347)	0.031	−0.93 ± 1.04^b^ (337)	−0.78 ± 1.01^a,b^ (356)	−0.69 ± 1.04^a^ (347)	0.011
WLZ	−0.54 ± 1.00 (350)	−0.64 ± 1.02 (342)	−0.49 ± 1.02 (347)	0.168	−0.64 ± 1.02 (337)	−0.54 ± 1.00 (355)	−0.49 ± 1.02 (347)	0.168
Head circumference, cm	45.2 ± 1.32 (353)	45.3 ± 1.30 (342)	45.4 ± 1.30 (348)	0.271	45.2 ± 1.35 (340)	45.3 ± 1.27 (355)	45.4 ± 1.30 (348)	0.107
HCZ	−1.14 ± 0.88 (350)	−1.14 ± 0.92 (342)	−1.07 ± 0.85 (347)	0.506	−1.18 ± 0.94 (337)	−1.10 ± 0.86 (355)	−1.07 ± 0.85 (347)	0.243
MUAC	14.2 ± 1.04 (353)	14.1 ± 1.05(343)	14.3 ± 1.05 (348)	0.255	14.1 ± 1.05 (340)	14.2 ± 1.04 (356)	14.3 ± 1.05 (348)	0.073

1 Values are means ± SDs (*n* analyzed for the variable in question). Mean ± SD values that are in the same row and have different superscript letters are significantly different. *n* = total number of participants in the group in question. IFA: infants received no supplementation, and their mothers received 60 mg Fe plus 400 μg folic acid during pregnancy only. MMN: infants received no supplementation, and their mothers received an MMN capsule containing 18 vitamins and minerals (including 20 mg Fe) during pregnancy and for the first 6 mo postpartum. LNS: infants received LNSs for infants from 6 to 18 mo of age, and their mothers received LNSs for women with the same micronutrients as the MMN group plus 4 more minerals (calcium, phosphorus, potassium, and magnesium) as well as macronutrients during pregnancy and for the first 6 mo postpartum. All supplements were intended for daily consumption. Results are based on ANOVA (SAS PROC GLIMMIX). HCZ, head circumference *z* score; IFA, iron and folic acid; LAZ, length-for-age *z* score; LNS, small-quantity lipid-based nutrient supplement; MMN, multiple micronutrient; MUAC, midupper arm circumference; WAZ, weight-for-age *z* score; WLZ, weight-for-length *z* score.

2 *P* values compare the mean ± SD of all 3 groups with Tukey-Kramer adjustment for pairwise comparisons.

In **Table 3**, the unadjusted binary anthropometric outcomes for infants by 18 mo of age are presented. When data were analyzed by using intervention groups based on intended supplement at enrollment, the point estimates for the prevalence of stunting, underweight, and small head circumference (with the except of wasting) were lowest for the LNS group compared with the other 2 groups, although the differences were not statistically significant (overall *P* > 0.05 for each outcome). In the analyses that used intervention groups based on supplements received at enrollment, there was a significantly lower (overall *P* = 0.045) prevalence of stunting in the LNS group (8.9%) than in the IFA group (15.1%) but not in the MMN group (11.5%) by 18 mo of age, and the prevalence of underweight was lower (overall *P* = 0.025) in the LNS (10.7%) and MMN (10.1%) groups than in the IFA group (16.3%). As observed for the continuous outcomes, adjusting for selected covariates did not change these results (details not shown).

**TABLE 3:** Binary anthropometric outcomes by 18 mo of age of infants of women (*n* = 1320) who participated in a randomized trial of daily IFA (pregnancy only), MMN (pregnancy and lactation), and LNS (pregnancy, lactation, and infancy) supplementation in a semi-urban setting in Ghana, by intervention group^1^

	Intervention groups based on intended supplement at enrollment				Intervention groups based on supplements received at enrollment			
	IFA (*n* = 441)	MMN (*n* = 439)	LNS (*n* = 440)	*P*^2^	IFA (*n* = 441)	MMN (*n* = 439)	LNS (*n* = 440)	*P*^2^
Stunting, LAZ <−2	13.7 (10.5, 17.7) [350]	12.9 (9.7, 16.9) [342]	8.9 (6.4, 12.4) [347]	0.118	15.1 (11.7, 19.4)^b^ [337]	11.5 (8.6, 15.3)^a,b^ [355]	8.9 (6.4, 12.4)^a^ [347]	0.045
Underweight, WAZ <−2	11.1 (8.2, 14.9) [350]	15.2 (11.7, 19.4) [343]	10.7 (7.8, 14.4) [347]	0.145	16.3 (12.7, 20.7)^b^ [337]	10.1 (7.4, 13.7)^a^ [356]	10.7 (7.8, 14.4)^a,b^ [347]	0.025
Wasting, WLZ <−2	6.0 (3.9, 9.0) [350]	9.1 (6.4, 12.6) [342]	6.3 (4.2, 9.4) [347]	0.235	9.2 (6.5, 12.8) [337]	5.9 (3.9, 8.9) [355]	6.3 (4.2, 9.4) [347]	0.197
Small head circumference, HCZ <−2	15.4 (12.0, 19.6) [350]	16.4 (12.8, 20.7) [342]	14.1 (10.8, 18.2) [347]	0.712	17.8 (14.1, 22.3) [337]	14.1 (10.8, 18.1) [355]	14.1 (10.8, 18.2) [347]	0.302

1 Values are the percentage of participants whose response was “yes” for the outcome in question (95% CIs) [*n* analyzed for the outcome in question]. For each analysis scenario, values that are in the same row and have different superscript letters are significantly different at α = 0.05. *n* = total number of participants in the group in question. IFA group: infants received no supplementation, and their mothers received 60 mg Fe plus 400 μg folic acid during pregnancy only. MMN group: infants received no supplementation, and their mothers received an MMN capsule containing 18 vitamins and minerals (including 20 mg Fe) during pregnancy and for the first 6 mo postpartum. LNS group: infants received LNSs for infants from 6 to 18 mo of age, and their mothers received LNSs for women with the same micronutrients as the MMN group plus 4 more minerals (calcium, phosphorus, potassium, and magnesium) as well as macronutrients during pregnancy and for the first 6 mo postpartum. All supplements were intended for daily consumption. Results are based on logistic regression models (SAS PROC GLIMMIX). HCZ, head circumference *z* score; IFA, iron and folic acid; LAZ, length-for-age *z* score; LNS, small-quantity lipid-based nutrient supplement; MMN, multiple micronutrient; MUAC, midupper arm circumference; WAZ, weight-for-age *z* score; WLZ, weight-for-length *z* score.

2 *P* values compare all 3 groups with Tukey-Kramer adjustment for pairwise comparisons.

In the secondary 2-group analysis in which children in the combined IFA and MMN groups were compared with those in the LNS group, we also found greater mean length (0.61 cm; *P* = 0.001) and weight (210 g; *P* = 0.010) in the LNS group by 18 mo of age (**Supplemental Table 1**), which corresponded to greater mean LAZ (0.20; *P* = 0.002) and WAZ (16; *P* = 0.018), as well as lower risk of stunting (unadjusted RR = 0.67; 95% CI: 0.46, 0.99) (**Supplemental Table 2**). As with the 3-group comparison, we found no difference between the LNS group and the combined IFA and MMN groups in the other outcomes measured.

**FIGURE 2:**
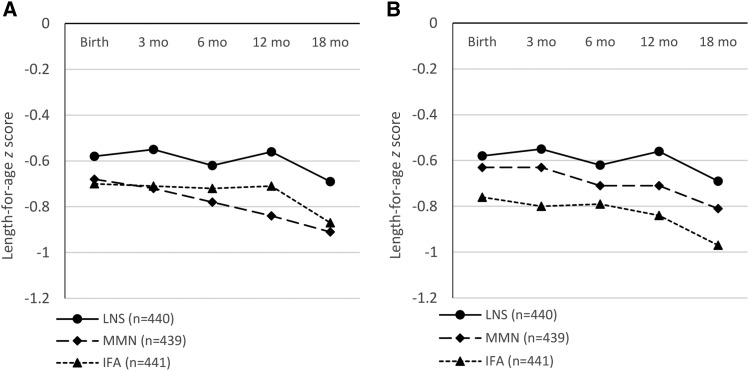
Length-for-age *z* scores from birth to 18 mo of age of infants of women (*n* = 1320) who participated in a randomized trial of daily IFA (pregnancy only), MMN (pregnancy and lactation), and LNS (pregnancy, lactation, and infancy) supplementation in a semi-urban setting in Ghana. (A) Analysis of groups based on intended supplement. Mean ± SD (*n*) at birth: −0.58 ± 0.97 (379), −0.68 ± 1.01 (388), and −0.70 ± 1.05 (386); at 3 mo: −0.55 ± 0.97 (341), −0.72 ± 0.99 (349), and −0.71 ± 1.02 (351); at 6 mo: −0.62 ± 1.04 (351), −0.78 ± 0.96 (355), and −0.72 ± 1.03 (347); at 12 mo: −0.56 ± 1.05 (331), −0.84 ± 1.03 (330), and −0.71 ± 1.02 (347); and at 18 mo −0.69 ± 1.01 (347), −0.91 ± 1.01 (342), and −0.87 ± 0.99 (350) for LNS, MMN, and IFA groups, respectively. (B) Analysis of groups based on supplements received at enrollment. Mean ± SD (*n*) at birth: −0.58 ± 0.97 (379), −0.63 ± 1.02 (386), and −0.76 ± 1.04 (388); at 3 mo: −0.55 ± 0.97 (341), −0.63 ± 1.02 (357), and −0.80 ± 0.98 (343); at 6 mo: −0.62 ± 1.04 (351), −0.71 ± 1.01 (355), and −0.79 ± 0.98 (347); at 12 mo: −0.56 ± 1.05 (331), −0.71 ± 1.05 (345), and −0.84 ± 1.00 (332); at 18 mo: −0.69 ± 1.01 (347), −0.81 ± 1.01 (355), and −0.97 ± 0.97 (337) for LNS, MMN, and IFA groups, respectively. IFA, iron and folic acid; LNS, small-quantity lipid-based nutrient supplement; MMN, multiple micronutrient.

**Table 4** shows that the changes in length, LAZ, and the other continuous anthropometric outcomes from birth to 18 mo of age, with the exception of WLZ, did not differ by group, whether in the analysis of groups based on the intended supplement at enrollment or in the analysis of groups based on the supplement received at enrollment. Mean ± SD WLZ change was greater in the IFA group than in the MMN group only in the analysis based on the intended supplement at enrollment. Adjustments for selected covariates did not alter these results (data not shown). **Figure 2**A, B shows that regardless of the 2 analytic scenarios the differences in mean LAZ between the 3 groups at birth were sustained throughout the first 18 mo of life.

**TABLE 4:** Change in continuous anthropometric outcomes from birth to 18 mo of age of infants of women (*n* = 1320) who participated in a randomized trial of daily IFA (pregnancy only), MMN (pregnancy and lactation), and LNS (pregnancy, lactation, and infancy) supplementation in a semi-urban setting in Ghana, by intervention group^1^

	Intervention groups based on intended supplement at enrollment				Intervention groups based on supplements received at enrollment			
Outcome variables	IFA (*n* = 441)	MMN (*n* = 439)	LNS (*n* = 440)	*P*^2^	IFA (*n* = 441)	MMN (*n* = 439)	LNS (*n* = 440)	*P*^2^
Length change, cm	30.8 ± 2.5 (337)	30.7 ± 2.5 (331)	31.0 ± 2.5 (334)	0.171	30.6 ± 2.4 (330)	30.8 ± 2.6 (338)	31.0 ± 2.5 (334)	0.147
LAZ change	−0.19 ± 0.99 (337)	−0.25 ± 1.01 (331)	−0.16 ± 0.98 (334)	0.493	−0.23 ± 0.99 (330)	−0.21 ± 1.01 (338)	−0.16 ± 0.98 (334)	0.641
Weight change, kg	6.8 ± 1.14 (339)	6.6 ± 1.1 (335)	6.8 ± 1.1 (334)	0.110	6.7 ± 1.1 (330)	6.8 ± 1.1 (344)	6.8 ± 1.1(334)	0.214
WAZ change	−0.05 ± 1.17 (339)	−0.23 ± 1.15 (335)	−0.19 ± 1.13 (334)	0.092	−0.13 ± 1.18 (330)	−0.15 ± 1.14 (344)	−0.19 ± 1.13 (334)	0.813
WLZ change	−0.05 ± 1.27^a^ (323)	−0.32 ± 1.36^b^ (318)	−0.25 ± 1.28^a,b^ (323)	0.025	−0.17 ± 1.35 (313)	−0.20 ± 1.30 (328)	−0.25 ± 1.28 (323)	0.735
Head circumference change, cm	11.5 ± 1.4 (338)	11.5 ± 1.3 (334)	11.4 ± 1.3 (331)	0.621	11.5 ± 1.3 (329)	11.5 ± 1.4 (343)	11.4 ± 1.3 (331)	0.764
HCZ change	−0.72 ± 1.08 (338)	−0.79 ± 1.03 (334)	−0.82 ± 0.99 (331)	0.419	−0.74 ± 1.04 (329)	−0.77 ± 1.07 (343)	−0.82 ± 0.99 (331)	0.594

1 Values are means ± SDs (*n* analyzed for the variable in question). Mean ± SD values in the same row that have different superscript letters are significantly different. *n* = total number of participants in the group in question. IFA group: infants received no supplementation, and their mothers received 60 mg Fe plus 400 μg folic acid during pregnancy only. MMN group: infants received no supplementation, and their mothers received an MMN capsule containing 18 vitamins and minerals (including 20 mg Fe) during pregnancy and for the first 6 mo postpartum. LNS group: infants received LNSs for infants from 6 to 18 mo of age, and their mothers received LNSs for women with the same micronutrients as the MMN group plus 4 more minerals (calcium, phosphorus, potassium, and magnesium) as well as macronutrients during pregnancy and for the first 6 mo postpartum. All supplements were intended for daily consumption. Results are based on ANOVA (SAS PROC GLIMMIX). HCZ, head circumference *z* score; IFA, iron and folic acid; LAZ, length-for-age *z* score; LNS, small-quantity lipid-based nutrient supplement; MMN, multiple micronutrient; MUAC, midupper arm circumference; WAZ, weight-for-age *z* score; WLZ, weight-for-length *z* score.

2 *P* values compare all 3 groups with Tukey-Kramer adjustment for pairwise comparisons.

### Effect modification

In prespecified tests for interaction of groups based on intended supplement at enrollment, child sex and maternal BMI, age, anemia status, GA at enrollment, and household food insecurity score did not modify the effect of the intervention on any of the anthropometric outcomes by 18 mo of age (all *P*-interaction > 0.10). In subgroup analyses stratified by the factors for which there were significant interactions (all *P*-interaction < 0.10), the differences in anthropometric outcomes by 18 mo of age between intervention groups were more pronounced for LAZ, weight, and WAZ among children of primiparous women; stunting among children of taller women and those with more education; wasting among children of women with more education, with a lower housing score, or enrolled in the dry season; and small head circumference among children of women with a higher household asset score (**Supplemental Table 3**).

When groups based on supplement actually received at enrollment were used, mean LAZ of infants was greater in the LNS group than in the MMN group among children of women with lower (but not higher) household asset score; the risk of stunting was lower in the LNS group than the in IFA or MMN group among taller (but not shorter) women; the risk of wasting was lower in the LNS group than in the IFA group among children of women with lower housing score and primiparous (but not multiparous) women; and the risk of small head circumference was lower in the LNS group than in the IFA group among children of women with higher household assets score and primiparous (but not multiparous) women (Supplemental Table 3).

### Occurrence of SAEs

Throughout the study (**Table 5**), we recorded 223 SAEs involving 203 women and 252 SAEs involving 211 children. For women, apart from the 5 deaths, 35 miscarriages, and 27 stillbirths reported previously (22), there was a total of 153 hospitalizations and 1 life-threatening condition that required an immediate hospital visit. For children, the SAEs consisted of 27 deaths, 223 hospitalizations, and 2 cases of congenital abnormalities. None of the recorded SAEs were judged by an independent consulting physician as likely to be caused by the intervention. The main cause of hospitalization was malaria that occurred either alone or in combination with other infections. All the different types of SAEs were evenly distributed across the 3 groups in both the analysis of groups based on intended supplement at enrollment and the analysis of groups based on supplements received at enrollment.

**TABLE 5:** Occurrence of serious adverse events in women (*n* = 1320) and their infants (*n* = 1228) who participated in a randomized trial of daily IFA (pregnancy only), MMN (pregnancy and lactation), and LNS (pregnancy, lactation, and infancy) supplementation in a semi-urban setting in Ghana, by intervention group^1^

	Intervention groups based on intended supplement at enrollment				Intervention groups based on supplements received at enrollment			
	IFA (*n* = 441)	MMN (*n* = 439)	LNS (*n* = 440)	*P*^2^	IFA (*n* = 441)	MMN (*n* = 439)	LNS (*n* = 440)	*P*^2^
Women								
Total SAEs^3^	70	75	78	0.80^4^	76	69	78	0.76^4^
Women who experienced SAEs	63/441 (14.3)	71/439 (16.2)	69/440 (15.7)	0.72	69/441 (15.6)	65/439 (14.8)	69/440 (15.7)	0.92
Hospitalizations	44	50	59	0.34^4^	48	46	59	0.40^4^
Women who were hospitalized	39/441 (8.8)	49/439 (11.2)	54/440 (12.3)	0.25	45/441 (10.2)	43/439 (9.8)	54/440 (12.3)	0.45
Deaths	3/441 (0.7)	1/439 (0.2)	1/440 (0.2)	0.49	1/441 (0.2)	3/439 (0.7)	1/440 (0.2)	0.48
Women who required immediate hospital visit	0/441 (0.0)	0/439 (0.0)	1/440 (0.2)	1.00	0/441 (0.0)	0/439 (0.0)	1/440 (0.2)	1.00
Children (from delivery to 18 mo of age)								
Total SAEs	97	67	88	0.11^4^	91	73	88	0.38^4^
Children who experienced SAEs	78/411 (19.0)	57/408 (14.0)	76/409 (18.6)	0.11	70/408 (17.2)	65/411 (15.8)	76/409 (18.6)	0.58
Hospitalizations	90	58	75	0.07^4^	84	64	75	0.32^4^
Children who were hospitalized	73/411 (17.8)	48/408 (11.8)	65/409 (15.9)	0.05	64/408 (15.7)	57/411 (13.9)	65/409 (15.9)	0.67
Deaths	7/411 (1.7)	8/408 (2.0)	12/409 (2.9)	0.46	7/408 (1.7)	8/411 (1.9)	12/409 (2.9)	0.46
Children with congenital abnormalities	0/411 (0.0)	1/408 (0.2)	1/409 (0.2)	1.00	0/408 (0.0)	1/411 (0.2)	1/409 (0.2)	1.00

1 Values are the number of cases of the outcome variable in question or number of participants whose response was “yes” for the outcome variable in question/*n* analyzed for the outcome variable in question (percentage of participants analyzed for the outcome variable in question).* n* = total number of participants in the group in question. IFA group: infants received no supplementation, and their mothers received 60 mg Fe plus 400 μg folic acid during pregnancy only. MMN group: infants received no supplementation, and their mothers received an MMN capsule containing 18 vitamins and minerals (including 20 mg Fe) during pregnancy and for the first 6 mo postpartum. LNS group: infants received LNSs for infants from 6 to 18 mo of age, and their mothers received LNSs for women with the same micronutrients as the MMN group plus 4 more minerals (calcium, phosphorus, potassium, and magnesium) as well as macronutrients during pregnancy and for the first 6 mo postpartum. All supplements were intended for daily consumption. IFA, iron and folic acid; LNS, small-quantity lipid-based nutrient supplement; MMN, multiple micronutrient; SAE, serious adverse event.

2 *P* values were obtained from logistic regression unless otherwise indicated.

3 Total number of SAEs for women include miscarriages and stillbirths which were previously reported (22).

4 *P* values were obtained from ANOVA.

## DISCUSSION

We found that children in the LNS group had greater length, LAZ, weight, and WAZ than those in the IFA or MMN group by 18 mo of age, regardless of whether the groups were defined by intended supplement at enrollment or by the supplement actually received at enrollment. The proportion of children who were stunted by 18 mo of age was significantly lower in the LNS group (8.9%) than in the IFA group (15.1%) only when the groups were defined based on the supplement received at enrollment, although the same trend was evident when the groups were defined based on intended supplement at enrollment. In the sensitivity analysis in which children in the LNS group were compared with those in the IFA and MMN groups combined, the differences in length, weight, and stunting prevalence were significant. The consistency in results between the 2 analytic scenarios as well as the sensitivity analysis suggests that our findings were not biased by the protocol violation. These findings support the hypothesis that in these semi-urban communities in Ghana, supplementation of women’s diet during pregnancy and the first 6 mo postpartum, and of their infants’ diet from 6 to 18 mo of age with SQ-LNSs increased attained size of children by 18 mo of age.

This study has several strengths, including using a fully randomized design, having active control groups, and blinding of participants and study workers to IFA or MMN group assignments. In addition, all anthropometrists were well trained and standardized (16, 32), and we undertook all efforts to ensure data quality. Study weaknesses include the inability to fully blind all study staff and participants to the supplementation allocation (because of the obvious differences between the IFA and MMN capsules and the SQ-LNS sachets), assessment of adherence to supplement intakes via maternal reporting rather than through direct observation, and the exposure of 340 women in the IFA and MMN groups to unintended supplements during all or part of pregnancy. However, all anthropometrists and data analysts were fully blinded to the group assignments until analyses were completed. Also, the actual percentage of follow-up days during which the women in the IFA and MMN groups had the unintended exposure was relatively small (13%), and no women in the LNS group were exposed to any other supplement apart from the intended SQ-LNS. We therefore believe that the study weaknesses do not bias the finding of greater growth by 18 mo in the SQ-LNS group.

Two studies (20, 21, 33, 34) involved the use of LNSs in prenatal supplementation followed by postnatal supplementation or follow-up, with which we can compare our results. First is the iLiNS-DYAD trial in Malawi referred to above (20, 21), in which SQ-LNSs, evaluated by using the same design as this study, did not increase linear growth of children by 18 mo of age. As noted by one commentator (35), a major reason for the difference in results between the Malawi study (20, 21) and this one may be the study context. The context in Malawi was that of a rural area, where linear growth may have been restricted by factors such as a high prevalence of asymptomatic infections, environmental enteropathy, and short maternal stature. Further, ∼11−15% of women across the 3 groups in the Malawi study (20) were HIV positive at baseline, compared with no documented HIV infection in the present study. In Burkina Faso, prenatal supplementation with medium-quantity LNSs, compared with MMNs, increased birth length (33), but the increase was not sustained during the ensuing 12 mo postpartum, when there was no LNS supplementation of mothers or infants (34). Our results are somewhat consistent with those from Burkina Faso (33, 34), in that prenatal LNS supplementation increased fetal growth in both cases, albeit primarily among primiparous women in our study (22). However, in Ghana the growth differences at birth were sustained through 18 mo of age, although there was no widening of the growth difference between groups after birth.

It is notable that the 3 groups did not differ significantly in growth pattern between birth and 18 mo of age, and that the group differences by 18 mo reflected the differences that existed at birth. Child growth in utero and during the first 2 y after birth is known to be influenced by a myriad of factors (4), including genetic (36, 37) as well as environmental factors, such as nutrition (38), infections (39), indoor air pollution (40, 41), and exposure to toxins (42–44). In this Ghanaian population, the overall rate of stunting by 18 mo of age (∼12%) was quite low compared with other contexts, such as Malawi (21, 45) and Burkina Faso (46), and therefore there was little room for improvement. There was no linear-growth faltering in the LNS group in this study between birth and 12 mo and only a slight decline in LAZ between 12 and 18 mo, which is very different from the pattern in many low-income populations (13). Earlier results from Ghana (16), Haiti (47), Malawi (17), and Burkina Faso (46) suggested that supplementation of infants’ diet with LNSs either alone (16, 17, 47) or in combination with other interventions (46) increased linear growth or reduced stunting in children, although the results from Haiti have been questioned (48), and in another study in Malawi (45), supplementation of infants’ diets from 6 to 18 mo of age did not prevent growth faltering. The lack of differences in postnatal growth in the current study suggests that the provision of SQ-LNSs to the children from age 6 to 18 mo had little impact in this context, but without a group that received only maternal SQ-LNSs, we cannot say what the growth pattern would have been without that component of the intervention.

In Ghana, like many developing country settings, inadequate maternal and infant nutrient intake is thought to be a major contributor to poor linear growth (49), and therefore it is logical that the increased supply of energy and micronutrients from SQ-LNSs would reduce the rate of or prevent growth faltering in our sample. Evidence from another iLiNS trial in Malawi (45) showed that the provision of LNSs did not decrease breast milk intake at 9 mo of age (50), whereas it increased the energy intake from complementary foods (51). At endline (18 mo of age) in all the 4 iLiNS trials (21, 22, 45, 46), the provision of LNSs did not change breastfeeding practices, whereas it increased the frequency of feeding with complementary foods in 2 of the trials (21, 46) in which data for this indicator were collected (52). These iLiNS results are consistent with several other studies that have shown no reduction in breast milk intake or in complementary food intake with the provision of LNSs (16, 53–55). Also, this study was conducted in a semi-urban area, where nearly all participants had access to potable water, electricity, and health care (e.g., through the National Health Insurance Scheme), and thus the negative impact of infections and environmental enteropathy on linear growth (39) may have been less of a factor than in other settings, making it more likely to see an effect of a nutritional intervention on child growth. In a peri-urban area in Peru (56), where stunting at 18 mo was also relatively low, a nutrition education intervention reduced stunting at 18 mo of age compared with a control group. Our results and those from the Peru study (56) suggest that nutrition-only interventions may be effective at increasing linear growth and reducing stunting in settings in which the overall prevalence of stunting is relatively low and access to health care is relatively good, whereas integrated interventions that target multiple causes of stunting may be necessary in other contexts (1).

The prespecified tests of interaction showing statistically significant effect modification of children’s anthropometric outcomes at 18 mo of age by various maternal and household factors warrant some discussion. It is possible that some of these interactions were significant by chance, because of multiple testing (57). Nonetheless, the finding (in both analytic scenarios) of a greater effect of SQ-LNS consumption on stunting among children of taller (compared with shorter) women is consistent with our observation that linear-growth responses to nutrition-only interventions are more likely in subgroups who have fewer constraints on child growth. Short maternal stature may be one such constraint that is difficult to overcome with such interventions. Although we observed a greater impact of the prenatal component of this intervention on birth size among primiparous than among multiparous women (22), primiparity did not consistently modify the effect of the intervention on attained size by 18 mo. This was because there was somewhat less postnatal growth faltering in the LNS group than in the other 2 groups among the infants of multiparous women but not among infants of primiparas (data not shown), so in both parity groups the SQ-LNS children ended up with greater attained size by 18 mo when compared with the other groups.

We conclude that prenatal and postnatal provision of SQ-LNSs may be effective for improving linear growth of children in similar settings. In populations with a greater burden and more complex etiology of stunting, the impact of SQ-LNSs should be tested more broadly in the context of programmatic initiatives that integrate nutrition into more comprehensive strategies to reduce stunting.

## Footnotes

7 Abbreviations used: EFA, essential fatty acid; GA, gestational age; HCZ, head circumference-for-age *z* score; IFA, iron and folic acid; iLiNS, International Lipid-Based Nutrient Supplements; LAZ, length-for-age *z* score; LNS, lipid-based nutrient supplement; MMN, multiple micronutrient; MUAC, midupper arm circumference; SAE, serious adverse event; SQ-LNS, small-quantity, lipid-based nutrient supplement; WAZ, weight-for-age *z* score; WLZ, weight-for-length *z* score.
